# Low Serum Levels of Vitamins A, D, and E Are Associated with Recurrent Respiratory Tract Infections in Children Living in Northern China: A Case Control Study

**DOI:** 10.1371/journal.pone.0167689

**Published:** 2016-12-09

**Authors:** Xuguang Zhang, Fengshu Ding, Huaining Li, Wenfeng Zhao, Hong Jing, Yageng Yan, Yanping Chen

**Affiliations:** 1 Department of Clinical Nutrition, Harbin Children's Hospital, Harbin, China; 2 Department of Clinical Laboratory, Harbin Children's Hospital, Harbin, China; 3 Department of Clinical Nutrition, The first Affiliated Clinical Hospital of Harbin Medical University, Harbin, China; Centre National de la Recherche Scientifique, FRANCE

## Abstract

**Background:**

This study aimed to investigate the association of serum concentrations of vitamin A, D, and E with recurrent respiratory tract infections (RRTIs).

**Methods:**

A total of 1200 children aged at 0.5–14 years were selected via a face-to-face survey in Harbin, China. Among the participants, 600 children with RRTIs comprised the symptomatic group (RRTI group), whereas 600 healthy children were used as controls (control group). Blood samples were collected to measure serum levels of vitamins A and E by HPLC; the serum level of 25-hydroxycholecalciferol (25(OH)D), was measured by HPLC-MS/MS.

**Results:**

Serum levels of vitamins A and E, as well as 25(OH)D, were significantly lower in the RRTI group than the control group. The conditional logistic regression model and the receiver-operating characteristic curve showed that the insufficiency or deficiency of vitamins A, D, and E was positively correlated with RRTI occurrence (*p* < 0.05).

**Conclusions:**

Low serum concentrations of vitamins A, D, and E were associated with RRTIs in children from northern China.

## Introduction

Respiratory tract infections (RTIs) are common diseases worldwide, especially in low- and middle-income countries; these infections are the major causes of high pediatric morbidity and mortality among children [1]. Recurrent RTIs (RRTIs) often prolong the recovery period, increase the required health care, and cause more frequent complications, such as autoimmune disorders and septicemia [2]. Previous studies showed that the incidence of RRTIs among children in northern China ranged from 17.8% to 18.7% in 2011 [3]. RRTIs affect pediatric health and may cause economic and social problems, which are caused by treatment and hospitalization costs, increased school absenteeism, and the loss of working days among parents and caregivers of affected individuals [4].

RRTIs are attributed to deficiencies in local or systemic host defense, as well as pulmonary disorders caused by structural, functional, or environmental factors [5]. The environmental factors involved in RRTIs include the nutrient deficiency, such as that of zinc, heavy metal pollution, a history of allergies, and the maternal health during pregnancy; these factors may affect the resistance of children against pathogenic microorganisms [3, 5].

Vitamins A, D, and E are multifunctional lipid-soluble compounds that are necessary for human health. Vitamin A is an essential nutrient for normal visual function and the maintenance of cell function for growth, epithelial integrity, immunity, and reproduction [6, 7]. Vitamin D is essential for healthy bones and may play roles in muscle function and the immune system. Vitamin E has been recognized as an essential nutrient for reproduction since 1922. This vitamin is a major lipid-soluble antioxidant of all cellular membranes, which prevents chronic diseases associated with oxidative stresses [8, 9]. A deficiency of these vitamins may cause nyctalopia, rickets, and ataxia, as well as increase the sensitivity to diarrheal diseases, type 1 diabetes, tuberculosis, and coronary heart disease [10–13].

Numerous studies have focused on identifying the association between the inadequate concentrations of vitamins A, D, and E and the incidence of RTIs in children [14, 15]. However, previous results have been contentious; no evidence is available to prove these hypotheses. Therefore, the roles of vitamins A, D, and E in children with RTIs and RRTIs should be clarified.

In this study, we evaluated the correlations of serum levels of vitamins A, D, and E with the occurrence of RRTIs in children from northern China. Data showed that the insufficient or deficient levels of vitamins A, D, and E were positively correlated with RRTIs. The findings provide insights into the roles of these fat-soluble vitamins in RRTIs and support the need for further studies on the prevention and treatment of RRTIs.

## Materials and Methods

### Ethics statement

This research project was approved by the Ethics Committee of the Harbin Children's Hospital on January 1, 2015 [No. HRYLL201501]. The patient information, such as the name, date of birth, height, weight, and time of respiratory infections within one year, were obtained from the parents or guardians of all participants. Each item was well explained before consent was written.

### Subjects and clinical assessment

A total of 1200 individuals were selected from the outpatient department at Harbin Children's Hospital (Heilongjiang, China) via a face-to-face questionnaire. The period of enrollment ran from April 2015 to May 2016. Assessment was based on the diagnostic criteria for RRTIs according to the consensus guidelines published by the Breathing Group of the Pediatrics Branch of the Chinese Medical Association [3] (Table 1), 600 of the 1200 individuals suffered from RRTIs but had no RTI symptoms upon enrollment; these individuals were placed in the RRTI group. Based on a case–control design, the other 600 healthy subjects, whose ages and genders were matched with the RRTI group, were used as asymptomatic controls and referred to as the control group. The exclusion criteria for both groups were preterm births, respiratory tract malformation, the presence of circulatory, neural, urinary, hematological, and digestive diseases, and the incidence of RTIs upon enrollment.

**Table 1:** Diagnostic Criteria of RRTIs.

Age (years)	Recurrent respiratory infections (times/year)	Recurrent bronchitis (times/year)	Recurrent pneumonia (times/year)
0–2	7	3	2
3–5	6	2	2
6–14	5	2	2

The height, weight, white blood cell count (WBC), and hemoglobin count (HGB), as well as the levels of vitamins A, D, and E, were measured in the survey. An anthropometric calculator (World Health Organization; http://www.who.int/en/) was used to determine the body mass index (BMI) for age.

### Quantification of serum levels of vitamins A, D, and E

Serum levels of vitamins A and E were determined by HPLC (LC-20AD; Shimadzu, Japan). By contrast, serum levels of vitamin D as 25-hydroxycholecalciferol (25(OH)D) were determined by HPLC-MS/MS (API3200; AB Sciex, USA).

### Statistical analysis

SPSS 16 (PASW Statistics 16) was used for the statistical analyses. Median and interquartile ranges were used to measure the central tendency and dispersion, respectively; the Wilcoxon’s rank sum test was used for abnormally distributed variables. Quantitative data were expressed as means ± SD and analyzed for the differences between the RRTI and control groups by the *t*-test with normal distribution. The chi-squared test was used to compare differences in the distribution of qualitative variables between groups. Values with *p* < 0.05 were considered significant for all statistical tests. Logistic regression was used to determine odds ratios (OR) and 95% confidence intervals (CI) to evaluate the association of RRTIs with the serum levels of vitamins A and E, as well as that of 25(OH)D. The receiver-operating characteristic (ROC) curve was used to summarize sensitivity and specificity across a range of cut-off points for a continuous predictor.

## Results

The ages and genders in the RRTI (*n* = 600) and control (*n* = 600) groups were matched. The median ages were 4.00 years old in both groups, with the interquartile range at 1.52–6.81 and 1.50–6.75 years in the RRTI and control groups, respectively (*Z* = 0.897, *p* = 0.370). The number of males and females was 234 (39%) and 366 (61%), respectively, in the RRTI and control groups.

The BMI-for-age is one of the indices used to express the development and health status in children; its values were 0.32 ± 1.21 and 0.35 ± 1.06 in the RRTI and control groups, respectively. The WBC and HGB levels were normal at (7.08 ± 1.94)×10^9^/L and 122.10 ± 8.33 g/L in the RRTI group and (7.26 ± 1.89)×10^9^/L and 122.94 ± 8.18 g/L in the control group). No significant differences were observed between the two groups for the BMI-for-age, as well as the WBC and HGB levels (*p* < 0.05; Table 2).

**Table 2:** BMI-for-age, with WBC and HGB levels.

Parameter	RRTI group (*n* = 600) (mean ± SD)	Control group (*n* = 600) (mean ± SD)	*p-*value*
BMI-for-age	0.32 ± 1.21	0.35 ± 1.06	>0.05
WBC [×10^9^/L]	7.08 ± 1.94	7.26 ± 1.89	>0.05
HGB [g/L]	122.10 ± 8.33	122.94 ± 8.18	>0.05

WBC, white blood cells; HGB, hemoglobin; BMI, body mass index.

*p < 0.05 shows a significant difference between the two groups

### Serum concentrations of vitamins A and E, as well as 25(OH)D, and their insufficiency or deficiency in the control and RRTI groups

The serum levels of vitamins A and E, as well as 25(OH)D, were 0.28 ± 0.07 mg/L, 20.52 ± 11.04 ng/mL, and 8.14 ± 2.32 mg/L in the control group, respectively, but these levels were 0.34 ± 0.07 mg/L, 29.11 ± 10.59 ng/mL, and 9.72 ± 2.56 mg/L, in the RRTI group. Statistical analysis showed that the levels of all three vitamins were significantly lower in the RRTI group compared with the control group (Table 3). The incidence of insufficiency or deficiency for vitamins A, E, and D was 24.33%, 8.17%, and 19.33% in the control group but increased to 63.00%, 33.83%, and 56.50% in the RRTI group, respectively. Significant differences were noted in the incidence of insufficiency or deficiency of vitamins A, D, and E between the two groups (*p* < 0.05; Table 4). These data suggested that low levels of vitamins A, D, and E may be associated with RRTIs.

**Table 3:** Vitamin A, D, and E concentrations.

Parameter	RRTI group (*n* = 600) (mean ± SD)	Control group (*n* = 600) (mean ± SD)	*p-*value*
Vitamin A (mg/L)	0.28 ± 0.07	0.34 ± 0.07	<0.001
Vitamin D (ng/mL)	20.52 ± 11.04	29.11 ± 10.59	<0.001
Vitamin E (mg/L)	8.14 ± 2.32	9.72 ± 2.56	<0.001

*p < 0.05 shows significant difference between two groups

**Table 4:** Incidences of insufficiencies or deficiencies of vitamins A, D, and E.

Parameter	RRTI group (*n* = 600) *n* (%)	Control group (*n* = 600) *n* (%)	*p-/Z*-value*
Vitamin A sufficient	222 (37.00)	454 (75.67)	
vitamin A insufficiency	314 (52.33)	134 (22.33)	
Vitamin A deficiency	64 (10.67)	12 (2.00)	<0.001
Vitamin D sufficient	261 (43.50)	484 (80.67)	
Vitamin D insufficiency	105 (17.50)	83 (13.83)	
Vitamin D deficiency	221 (36.83)	32 (5.33)	
Vitamin D severe sufficiency	13 (2.17)	1 (0.17)	<0.001
Vitamin E sufficient	397 (66.17)	551 (91.83)	
Vitamin E insufficiency	203 (33.83)	49/0 (8.17)	< 0.001
Vitamin E deficiency	0 (0)	0 (0)	

*p < 0.05 shows significant difference between two groups

### Correlation of vitamins A, D, and E levels with RRTIs

A logistic regression model was used to determine whether low levels of vitamins A, D, and E are associated with RRTIs. The OR value, which indicates correlation, was 1.848 (95% CI: 1.547–2.209) for vitamin A insufficiency, 2.025 (95% CI: 1.504–2.727) for vitamin A deficiency, 1.570 (95% CI: 1.239–1.988) for vitamin D insufficiency, 2.432 (95% CI: 2.010–2.942) for vitamin D deficiency, 1.957 (95% CI: 1.109–3.455) for significant vitamin D reduction, and 1.495 (95% CI: 1.249–1.789) for vitamin E insufficiency (Table 5). All OR values significantly increased (*p* < 0.05) with the increasing deficiency, except for the OR of significant vitamin D reduction. These results statistically confirmed that lower levels of vitamins A, D, and E are positively correlated with RRTIs occurrence.

**Table 5:** Associated factors screened in logistic regression model.

Parameter	B	*p*-value	OR value	95% CI of OR value	
				Lower	Upper
Vitamin A insufficiency	0.614	<0.001	1.848	1.547	2.209
Vitamin A deficiency	0.706	<0.001	2.025	1.504	2.727
Vitamin D insufficiency	0.451	<0.001	1.570	1.239	1.988
Vitamin D deficiency	0.889	<0.001	2.432	2.010	2.942
Vitamin D significant reduction	0.672	0.020	1.957	1.109	3.455
Vitamin E insufficiency	0.402	<0.001	1.495	1.249	1.789

### Sensitivity and specificity of the correlation between levels of vitamins A, D, and E with incidence of RRTIs

**Fig 1:**
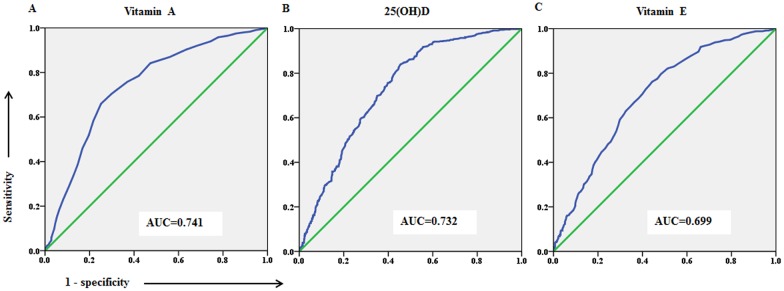
ROC curve for the predictive model of RRTIs with serum levels of vitamin A, 25(OH)D, and vitamin E. (a) Vitamin A, (b) 25(OH)D, and (c) vitamin E. The high level of agreement among the RRTIs and vitamin A, 25(OH)D, and vitamin E assays are indicated by the large AUC.

To further confirm the sensitivity of the levels of vitamins A, D, and E to RRTIs, a predicted threshold value was calculated using ROC curve. Vitamin A, 25(OH)D, and vitamin E showed area-under-the-curve (AUC) values of 0.741 (95% CI: 0.713–0.769; Fig 1A), 0.732 (95% CI: 0.704–0.760; Fig 1B), and 0.699 (95% CI: 0.670–0.729; Fig 1C), respectively, when asymptomatic individuals were used as controls. The cut-off values of vitamin A, 25(OH)D, and vitamin E were 0.315 mg/L, 19.45 ng/mL, and 7.85 mg/L, respectively, as the optimal cut-off values. As defined by the sum of maximum sensitivity and specificity, compared with the control values, vitamin A showed a sensitivity at 66.00% and specificity at 74.83%, 25(OH)D showed a sensitivity at 84.00% and specificity at 54.50%, and vitamin E showed a sensitivity at 76.20% and specificity at 55.70%.

## Discussion

Abnormal levels of micronutrients, including vitamins and minerals, have been associated with several medical conditions, as well as the increased risk of developing upper and lower RTIs. These RTIs and RRTIs are the major causes of pediatric morbidity and mortality [16, 17]. Numerous studies have focused on the identification of the association between inadequate serum concentrations of vitamins A, D, and E with the occurrence of RTIs in children [18]. However, the results remain controversial. Moreover, no evidence shows the relationship between fat-soluble vitamins and RRTIs in children. Therefore, better understanding of these fat-soluble vitamins is needed to prevent and treat RTIs and RRTIs among children. The present study demonstrated that lower serum levels of vitamins A, D, and E are associated with RRTIs for the first time. The study provides evidence for the further study of these vitamins to prevent and treat RRTIs.

Immunoglobulin A (IgA) is the primary antibody secreted into mucosal cavities as the first line of defense in [19]. The salivary IgA concentration in humans is related to upper RTIs [20]. Previous studies demonstrated that vitamin A plays an important role in T-cell differentiation, as well as IgA switching and production [21–23]. Mucin glycoproteins are the major components of airway mucus to provide a protective barrier against pathogenic agents. Airway mucins are mainly produced by the goblet and submucosal gland cells [24]. Vitamin A deficiency decreases the induction of mucin gene expression and weakens the lymphocyte proliferation response to pathogens [25, 26]. Our data showed that serum levels of vitamin A were significantly lower in RRTI group. Concurrently, the lower serum level of vitamin A is positively associated with RRTIs. The immune function of vitamin A may contribute to its role in RRTIs. The cut-off values suggested that vitamin A levels lower than 0.315 mg/L may be a risk factor for RRTIs. The data also suggested that normal serum levels of vitamin A (0.3 mg/L) [27] may not be enough to prevent RRTIs among children in northern China. The intake of vitamin A-enriched foods or routine supplements may be required to keep vitamin A serum levels above normal levels for individuals with RRTIs or the RRTI-susceptible population in northern China.

Vitamin D can boost the immune system by modulating innate and adaptive immunity and by regulating the inflammatory cascade [28, 29]. Vitamin D2 (ergocalciferol) and vitamin D3 (cholecalciferol) originate from food, such as liver and milk, or are synthesized by exposure to ultraviolet radiation B (UVB) [30]. Approximately 80% to 90% of the vitamin D level is dependent on the skin synthesis pathway; people who live in the northern latitudes, particularly during winter, probably do not synthesize adequate levels of vitamin D from sunlight [31]. Our data showed that the serum levels of 25(OH)D were lower in the RRTI group. The lower serum level of 25(OH)D is positively associated with RRTIs among children living in northern China, thereby suggesting that vitamin D may be important for preventing RRTIs because of its immune function. The OR value of vitamin D-deficient individuals was higher than that of insufficient individuals, thereby indicating the higher risk of RRTIs with lower serum levels of 25(OH)D. However, the OR values in individuals with significant vitamin D deficiency were lower than those observed for vitamin-deficient individuals in the present study. This trend may be attributed to the significantly low number of vitamin D-deficient individuals in our study. The cut-off value suggested that a 25(OH)D level lower than 19.45 ng/mL may be a risk factor for RRTIs; consequently, a normal serum level of 25(OH)D (20 ng/mL) [32] prevents RRTIs. Children living in northern latitudes lack sunlight; thus, vitamin D supplements and more sun exposure are needed to prevent RRTIs during winter.

The most recognized role of vitamin E is its function as a lipid-soluble antioxidant for preserving cell membranes, including immune cell membranes, to protect against oxidative damage related to the high metabolic activity and high polyunsaturated fatty acid content in these cells [33, 34]. Vitamin E is also an important immune regulator. To determine the role of vitamin E in RRTIs, we evaluated its relationship with the occurrence of RRTIs. No vitamin E-deficient subject was found in the present study. However, data showed that vitamin E serum levels were lower in RRTIs. A lower vitamin E serum level is positively associated with RRTIs in children, thereby indicating that sufficient vitamin E intake may help prevent the induction of RRTIs in children. The cut-off value suggested that a vitamin E serum level lower than 7.85 mg/L may be a risk factor for RRTIs, whereas vitamin E serum levels higher than the normal value (7 mg/mL) [35, 36] may prevent RRTIs in children.

This study has several limitations. First, the present study is prone to recall, selection, and measurement bias, which is a similar problem of other case–control trials. The possibility of subject selection bias, which may occur when subjects volunteer to join a study, cannot be disregarded. However, different response rates were not observed among the cases and controls; the different groups did not seem to have different levels of health consciousness.

Second, C-reactive protein (CRP) was detected as the inflammation indicator in the present study. Inflammation was defined as a CRP level >5 mg/L. However, given our limited laboratory, the lowest detectable limit of CRP was 5 mg/L. The CRP of all the included children was less than 5 mg/L because no RTI symptoms were observed upon enrollment.

Third, data measured for a whole year in the present study suggested that a lower serum level of 25(OH)D is positively associated with RRTIs in children. However, the individual serum levels of fat-soluble vitamins, such as vitamin D, always changed with seasons because of the different times of exposure to UVB. Future studies should expand the sample size and focus on the relationship between RRTI occurrence and soluble vitamins during different seasons.

Our data suggested that fat-soluble vitamins may play a role in RRTIs. A deficiency of some vitamins, such as vitamin D, may be associated with RRTIs because the immune system function is reduced, thereby increasing the sensitivity to RRTIs. Other vitamins, such as vitamins A and E, may be associated with RRTIs by promoting immune system function, thereby increasing the resistance to RRTIs. However, this retrospective study cannot conclude the role of these vitamins in RRTIs because we cannot determine whether the vitamin levels influenced the occurrence of RRTIs or vice versa. The RRTI group may have taken less vitamins than the control group because of their recurrent illness. Thus, a prospective study should be conducted to confirm the role of these fat-soluble vitamins in RRTIs. In addition, the effects of a single dose or a combination of these vitamins on patients with RRTIs might be of interest.

## Supporting Information

S1 STROBE Checklist STROBE_checklist_v4_combined_PlosMedicine. (DOCX)
