# Supplementary material for: Low Serum Levels of Vitamins A, D, and E Are Associated with Recurrent Respiratory Tract Infections in Children Living in Northern China: A Case Control Study
Source: PLoS One. 2016 Dec 9;11(12):e0167689. doi: 10.1371/journal.pone.0167689 (PMC5147939; doi:10.1371/journal.pone.0167689)
Supplement: S1 STROBE Checklist — (DOCX) [file pone.0167689.s001.docx]

STROBE Statement—checklist of items that should be included in reports of observational studies

|  | Item No. | Recommendation | Page  No. | Relevant text from manuscript |
| --- | --- | --- | --- | --- |
| **Title and abstract** | 1 | (*a*) Indicate the study’s design with a commonly used term in the title or the abstract | 1-2 | A Case Control Study |
|  |  | (*b*) Provide in the abstract an informative and balanced summary of what was done and what was found | 2 | Blood samples were collected to measure serum levels of vitamins A and E by HPLC; the serum level of vitamin D, as 25-hydroxycholecalciferol (25(OH)D), was measured by HPLC-MS/MS.  Low serum concentrations of vitamins A, D, and E were associated with RRTIs in children from northern China. |
| Introduction | | | |  |
| Background/rationale | 2 | Explain the scientific background and rationale for the investigation being reported | 4 | Respiratory tract infections (RTIs) are common diseases worldwide, especially in low- and middle-income countries; these infections are the major causes of high pediatric morbidity and mortality among children………….. |
| Objectives | 3 | State specific objectives, including any prespecified hypotheses | 4 | In this study, we evaluated the correlations of serum levels of vitamins A, D, and E with the occurrence of RRTIs in children from northern China. Data showed that the insufficient or deficient levels of vitamins A, D, and E were positively correlated with RRTIs. The findings provide insights into the roles of these fat-soluble vitamins in RRTIs and support the need for further studies on the prevention and treatment of RRTIs. |
| Methods | | | |  |
| Study design | 4 | Present key elements of study design early in the paper | 5 | Based on a case-control design; RRTI group; control group; age and gender were matched. |
| Setting | 5 | Describe the setting, locations, and relevant dates, including periods of recruitment, exposure, follow-up, and data collection | 4-5 | A total of 1200 individuals were selected from the outpatient department at Harbin Children's Hospital (Heilongjiang, China) via a face-to-face questionnaire. The period of enrollment ran from April 2015 to May 2016. |
| Participants | 6 | (*a*) *Cohort study*—Give the eligibility criteria, and the sources and methods of selection of participants. Describe methods of follow-up  *Case-control study*—Give the eligibility criteria, and the sources and methods of case ascertainment and control selection. Give the rationale for the choice of cases and controls  *Cross-sectional study*—Give the eligibility criteria, and the sources and methods of selection of participants | 4 | 600 subjects of the 1200 individuals who suffered from RRTIs, but had no RTIs symptoms at enrollment, were used as RRTIs group. Based on a case-control design, other 600 healthy subjects, were used as asymptomatic control, referred to as control group. |
|  |  | (*b*) *Cohort study*—For matched studies, give matching criteria and number of exposed and unexposed  *Case-control study*—For matched studies, give matching criteria and the number of controls per case | 5 | age and gender were matched |
| Variables | 7 | Clearly define all outcomes, exposures, predictors, potential confounders, and effect modifiers. Give diagnostic criteria, if applicable | 4-5 | Assessment was based on the diagnostic criteria for RRTIs according to the consensus guidelines published by the Breathing Group of the Pediatrics Branch of the Chinese Medical Association (Table 1) |
| Data sources/ measurement | 8* | For each variable of interest, give sources of data and details of methods of assessment (measurement). Describe comparability of assessment methods if there is more than one group | 5-6 | The height, weight, white blood cell count (WBC), and hemoglobin count (HGB), as well as the levels of vitamins A, D, and E, were measured in the survey. An anthropometric calculator (World Health Organization; http://www.who.int/en/) was used to determine the body mass index (BMI) for age.  Serum levels of vitamins A and E were determined by HPLC (LC-20AD; Shimadzu, Japan). By contrast, serum levels of vitamin D as 25-hydroxycholecalciferol (25[OH]D) were determined by HPLC-MS/MS (API3200; AB Sciex, USA). |
| Bias | 9 | Describe any efforts to address potential sources of bias |  |  |
| Study size | 10 | Explain how the study size was arrived at |  |  |

Continued on next page

| Quantitative variables | 11 | Explain how quantitative variables were handled in the analyses. If applicable, describe which groupings were chosen and why |  |  |
| --- | --- | --- | --- | --- |
| Statistical methods | 12 | (*a*) Describe all statistical methods, including those used to control for confounding | 6 | Median and interquartile range; means ± SD; Wilcoxon's rank sum test; *t*-test; Chi-square test; Logistic regression; receiver-operating characteristic curve |
|  |  | (*b*) Describe any methods used to examine subgroups and interactions |  |  |
|  |  | (*c*) Explain how missing data were addressed |  |  |
|  |  | (*d*) *Cohort study*—If applicable, explain how loss to follow-up was addressed  *Case-control study*—If applicable, explain how matching of cases and controls was addressed  *Cross-sectional study*—If applicable, describe analytical methods taking account of sampling strategy |  |  |
|  |  | (*e*) Describe any sensitivity analyses |  | Logistic regression; receiver-operating characteristic curve |
| Results | | | | |
| Participants | 13* | (a) Report numbers of individuals at each stage of study—eg numbers potentially eligible, examined for eligibility, confirmed eligible, included in the study, completing follow-up, and analysed | 6,7 | The ages and genders in the RRTI (n = 600) and control (n = 600) groups were matched. The number of males and females was 234 (39%) and 366 (61%), respectively, in the RRTI and control groups. |
|  |  | (b) Give reasons for non-participation at each stage |  |  |
|  |  | (c) Consider use of a flow diagram |  |  |
| Descriptive data | 14* | (a) Give characteristics of study participants (eg demographic, clinical, social) and information on exposures and potential confounders | 4 | Heilongjiang, China |
|  |  | (b) Indicate number of participants with missing data for each variable of interest |  |  |
|  |  | (c) *Cohort study*—Summarise follow-up time (eg, average and total amount) |  |  |
| Outcome data | 15* | *Cohort study*—Report numbers of outcome events or summary measures over time |  |  |
|  |  | *Case-control study—*Report numbers in each exposure category, or summary measures of exposure | 7-8 | No significant differences were observed between the two groups for the BMI-for-age, as well as the WBC and HGB levels.  The incidence of insufficiency or deficiency for vitamins A, E, and D was 24.33%, 8.17%, and 19.33% in the control group but increased to 63.00%, 33.83%, and 56.50% in the RRTI group, respectively. Significant differences were noted in the incidence of insufficiency or deficiency of vitamins A, D, and E between the two groups. |
|  |  | *Cross-sectional study—*Report numbers of outcome events or summary measures |  |  |
| Main results | 16 | (*a*) Give unadjusted estimates and, if applicable, confounder-adjusted estimates and their precision (eg, 95% confidence interval). Make clear which confounders were adjusted for and why they were included | 9-10 | The OR value, which indicates correlation, was 1.848 (95% CI: 1.547–2.209) for vitamin A insufficiency, 2.025 (95% CI: 1.504–2.727) for vitamin A deficiency, 1.570 (95% CI: 1.239–1.988) for vitamin D insufficiency, 2.432 (95% CI: 2.010–2.942) for vitamin D deficiency, 1.957 (95% CI: 1.109–3.455) for significant vitamin D reduction, and 1.495 (95% CI: 1.249–1.789) for vitamin E insufficiency (Table 5). All OR values significantly increased (p < 0.05) with the increasing deficiency, except for the OR of significant vitamin D reduction. |
|  |  | (*b*) Report category boundaries when continuous variables were categorized |  |  |
|  |  | (*c*) If relevant, consider translating estimates of relative risk into absolute risk for a meaningful time period | 10 | The cut-off values of vitamin A, 25(OH)D, and vitamin E were 0.315 mg/L, 19.45 ng/mL, and 7.85 mg/L, respectively, as the optimal cut-off values. |

Continued on next page

| Other analyses | 17 | Report other analyses done—eg analyses of subgroups and interactions, and sensitivity analyses |  |  |
| --- | --- | --- | --- | --- |
| Discussion | | | | |
| Key results | 18 | Summarise key results with reference to study objectives | 11 | Numerous studies have focused on the identification of the association between inadequate serum concentrations of vitamins A, D, and E with the occurrence of RTIs in children. However, the results remain controversial. Moreover, no evidence shows the relationship between fat-soluble vitamins and RRTIs in children. |
| Limitations | 19 | Discuss limitations of the study, taking into account sources of potential bias or imprecision. Discuss both direction and magnitude of any potential bias | 13-14 | This study has several limitations. First……… |
| Interpretation | 20 | Give a cautious overall interpretation of results considering objectives, limitations, multiplicity of analyses, results from similar studies, and other relevant evidence | 14-15 | Our data suggested that fat-soluble vitamins may play a role in RRTIs. A deficiency of some vitamins, such as vitamin D, may be associated with RRTIs because the immune system function is reduced, thereby increasing the sensitivity to RRTIs. Other vitamins, such as vitamins A and E, may be associated with RRTIs by promoting immune system function, thereby increasing the resistance to RRTIs. However, this retrospective study cannot conclude the role of these vitamins in RRTIs because we cannot determine whether the vitamin levels influenced the occurrence of RRTIs or vice versa. The RRTI group may have taken less vitamins than the control group because of their recurrent illness. Thus, a prospective study should be conducted to confirm the role of these fat-soluble vitamins in RRTIs. In addition, the effects of a single dose or a combination of these vitamins on patients with RRTIs might be of interest. |
| Generalisability | 21 | Discuss the generalisability (external validity) of the study results |  |  |
| Other information | |  | | |
| Funding | 22 | Give the source of funding and the role of the funders for the present study and, if applicable, for the original study on which the present article is based |  |  |

*Give information separately for cases and controls in case-control studies and, if applicable, for exposed and unexposed groups in cohort and cross-sectional studies.

**Note:** An Explanation and Elaboration article discusses each checklist item and gives methodological background and published examples of transparent reporting. The STROBE checklist is best used in conjunction with this article (freely available on the Web sites of PLoS Medicine at http://www.plosmedicine.org/, Annals of Internal Medicine at http://www.annals.org/, and Epidemiology at http://www.epidem.com/). Information on the STROBE Initiative is available at www.strobe-statement.org.
